# Octocoral Mitochondrial Genomes Provide Insights into the Phylogenetic History of Gene Order Rearrangements, Order Reversals, and Cnidarian Phylogenetics

**DOI:** 10.1093/gbe/evu286

**Published:** 2014-12-24

**Authors:** Diego F. Figueroa, Amy R. Baco

**Affiliations:** ^1^Present address: Department of Biological Sciences, University of Texas, Brownsville, TX; ^2^Department of Earth, Ocean and Atmospheric Science, Florida State University

**Keywords:** Octocorallia, deep-sea corals, soft corals, cnidarian phylogenetics, gene rearrangement, substitution saturation

## Abstract

We use full mitochondrial genomes to test the robustness of the phylogeny of the Octocorallia, to determine the evolutionary pathway for the five known mitochondrial gene rearrangements in octocorals, and to test the suitability of using mitochondrial genomes for higher taxonomic-level phylogenetic reconstructions. Our phylogeny supports three major divisions within the Octocorallia and show that Paragorgiidae is paraphyletic, with *Sibogagorgia* forming a sister branch to the Coralliidae. Furthermore, *Sibogagorgia cauliflora* has what is presumed to be the ancestral gene order in octocorals, but the presence of a pair of inverted repeat sequences suggest that this gene order was not conserved but rather evolved back to this apparent ancestral state. Based on this we recommend the resurrection of the family Sibogagorgiidae to fix the paraphyly of the Paragorgiidae.

This is the first study to show that in the Octocorallia, mitochondrial gene orders have evolved back to an ancestral state after going through a gene rearrangement, with at least one of the gene orders evolving independently in different lineages. A number of studies have used gene boundaries to determine the type of mitochondrial gene arrangement present. However, our findings suggest that this method known as gene junction screening may miss evolutionary reversals.

Additionally, substitution saturation analysis demonstrates that while whole mitochondrial genomes can be used effectively for phylogenetic analyses within Octocorallia, their utility at higher taxonomic levels within Cnidaria is inadequate. Therefore for phylogenetic reconstruction at taxonomic levels higher than subclass within the Cnidaria, nuclear genes will be required, even when whole mitochondrial genomes are available.

## Introduction

Octocorals, a group of corals commonly known as sea fans, sea whips, sea pens, and soft corals, play a key role in forming structures in a number of habitats including shallow water reefs, deep seamounts, and submarine canyons (Genin et al. 1986; Hecker 1990; Stocks 2004). They act as hosts for a variety of invertebrates and fishes, including some key deep-water fisheries species (Genin et al. 1992; Jones et al. 1994; Rogers 1994; Probert et al. 1997; Stocks 2004; DeVogelaere et al. 2005; Leverette and Metaxas 2005; Mortensen and Buhl-Mortensen 2005; Baco 2007; Buhl-Mortensen et al. 2010; Roberts et al. 2010; Baillon et al. 2012).

Deep-sea corals are slow growing, long lived, and existing evidence suggests that many are recruitment limited (Grigg 1988; Krieger 2001; Roark et al. 2006, 2009; Sun et al. 2010). Thus, they are very vulnerable to anthropogenic impacts and slow to recover from them (Williams et al. 2010). Anthropogenic activities that are known or likely to have large impacts on octocorals include fisheries (Koslow et al. 2001; Clark and Rowden 2009), deep-sea mining for cobalt-rich manganese crusts (Hein 2002; Hein et al. 2009), and climate change and ocean acidification (Guinotte et al. 2006).

Recent reviews of seamount fauna and deep-sea corals have concluded that the global deficiency of scientific expertise in morphological taxonomy is a significant impediment to the understanding of deep-sea coral diversity, coral biogeography, conservation, and seamount ecology (Morgan et al. 2006; Parrish and Baco 2007; Rogers et al. 2007). Likewise, in the past decade, molecular phylogenetic analyses of the anthozoan subclass Octocorallia have shown that the current taxonomic classification of these organisms, based on morphology, needs to be revised (Berntson et al. 2001; Sanchez et al. 2003; McFadden et al. 2006, 2010; Herrera et al. 2010; Brockman and McFadden 2012).

Until recently, the majority of phylogenetic analyses of octocorals have been based on a few mitochondrial genes or nuclear genes or a combination of both (Berntson et al. 2001; Sanchez et al. 2003; McFadden et al. 2006, 2010; Herrera et al. 2010); but recent studies are increasingly using whole mitochondrial genomes, revealing five different gene orders in octocorals (Brugler and France 2008; Uda et al. 2011; Brockman and McFadden 2012; Figueroa and Baco 2014). One of these gene orders is shared by most octocorals, while the other four alternative orders are only found within one of the three major clades of Octocorallia. Therefore, the widespread phylogenetic distribution of this gene order has led to the assumption that it represents the ancestral arrangement in octocorals (Brugler and France 2008; Uda et al. 2011; Brockman and McFadden 2012; Figueroa and Baco 2014). Whole mitochondrial genomes, which in octocorals contain 14 protein-coding genes, provide better resolution of the tree topology in these organisms (Uda et al. 2011). In general, molecular phylogenetic studies agree with the three major clades proposed by McFadden et al. (2006) based on sequences from two mitochondrial genes (*nad2* and *mutS*). One of these major clades is composed of the scleraxonians Coralliidae and Paragorgiidae and the alcyoniina *Anthomastus*, along with several other genera mostly belonging to the family Alcyoniidae (McFadden et al. 2006; Brockman and McFadden 2012; Figueroa and Baco 2014). These three families are among the most abundant octocoral families in the deep sea (Baco 2007) and thus improving their taxonomy is a high priority.

Thus the goal of our study was to improve our understanding of the relationships within this *Anthomastus*–*Corallium* clade, as well as the evolution of the gene orders within this clade. We sequenced the whole mitochondrial genome of two morphospecies of *Anthomastus* and the paragorgiid *Sibogagorgia cauliflora*, all three presumably members of McFadden et al.’s (McFadden et al. 2006) *Anthomastus*–*Corallium* clade. We also sequenced the whole mitochondrial genome of the primnoid *Narella hawaiinensis*, a member of McFadden et al. (2006) Calcaxonia–Pennatulacea clade is the sister branch to the *Anthomastus*–*Corallium* clade (McFadden et al. 2006; Brockman and McFadden 2012; Figueroa and Baco 2014).

In the process of examining the phylogenetic relationships among these families, we also have the opportunity to gain a better understanding of the utility of whole mitochondrial genomes for unraveling phylogenetics at higher taxonomic levels within the Cnidaria. Recent phylogenetic reconstructions based on whole mitochondrial genomes have suggested that Anthozoa is a paraphyletic group, with Octocorallia branching as a sister clade to the Medusozoa and not the Hexacorallia (Shao et al. 2006; Kayal and Lavrov 2008; Lavrov et al. 2008; Park et al. 2012; Kayal et al. 2013). This observation disagrees with current morphological classification and with phylogenetic reconstructions based on nuclear markers, which strongly support a monophyletic Anthozoa comprised of the Octocorallia and Hexacorallia (France et al. 1996; Odorico and Miller 1997; Berntson et al. 1999; Won et al. 2001; Collins 2002; Daly et al. 2007).

Thus another goal of our analysis is to use the newly sequenced mitochondrial genomes from recently collected specimens of Octocorallia in conjunction with mitochondrial genomes found in GenBank for other Anthozoa, Medusozoa, and Porifera for phylogenetic analyses at three different taxonomic levels: Within subclass Octocorallia, within class Anthozoa, and within the phylum Cnidaria. Thus, phylogenetic analyses were used to achieve three main objectives: 1) To elucidate the internal topology of the *Anthomastus*–*Corallium* clade, 2) to test the robustness of the phylogeny of Octocorallia proposed by McFadden et al. (2006), and 3) to test the suitability of mitochondrial genomes to be used in higher order phylogenetic reconstructions within Cnidaria.

## Materials and Methods

### Collections

For this study, we used four octocoral specimens: Two distinct morphotypes of the genus *Anthomastus* (one collected from Necker Ridge in the northern Central Pacific and a second morphotype from Derickson Seamount, just south of the Aleutian Islands); a specimen of *S. cauliflora* (also from Derickson Seamount); and a specimen of *N. hawaiinensis* (collected from Pioneer Bank in the Northwestern Hawaiian Islands). Samples from Hawaii and Necker were collected using the Pisces IV or V submersible, and from Derickson using the ROV Jason II. Corals were placed in insulated bioboxes for return to the surface and subsamples were frozen at −80 °C. The remainder of each specimen was deposited at the Smithsonian. United States National Museum (USNM)#s for each specimen are listed in table 1.

**Table 1:** Specimens used in this Study

Subphylum	Subclass	Species	USNM No.	Genbank Acession No.	Sequence From
Anthozoa	Hexacorallia	*Acropora tenuis*		NC_003522	GenBank
Anthozoa	Hexacorallia	*Agaricia humilis*		NC_008160	GenBank
Anthozoa	Hexacorallia	*Anacropora matthai*		NC_006898	GenBank
Anthozoa	Hexacorallia	*Astrangia* sp. JVK-2006		NC_008161	GenBank
Anthozoa	Hexacorallia	*Chrysopathes formosa*		NC_008411	GenBank
Anthozoa	Hexacorallia	*Colpophyllia natans*		NC_008162	GenBank
Anthozoa	Hexacorallia	*Discosoma* sp. CASIZ 168915		NC_008071	GenBank
Anthozoa	Hexacorallia	*Discosoma* sp. CASIZ 168916		NC_008072	GenBank
Anthozoa	Hexacorallia	*Euphyllia ancora*		NC_015641	GenBank
Anthozoa	Hexacorallia	*Fungiacyathus stephanus*		NC_015640	GenBank
Anthozoa	Hexacorallia	*Goniopora columna*		NC_015643	GenBank
Anthozoa	Hexacorallia	*Lophelia pertusa*		NC_015143	GenBank
Anthozoa	Hexacorallia	*Madracis mirabilis*		NC_011160	GenBank
Anthozoa	Hexacorallia	*Metridium senile*		NC_000933	GenBank
Anthozoa	Hexacorallia	*Metridium senile*		NC_000933	GenBank
Anthozoa	Hexacorallia	*Montastraea annularis*		NC_007224	GenBank
Anthozoa	Hexacorallia	*Montastraea faveolata*		NC_007226	GenBank
Anthozoa	Hexacorallia	*Montastraea franksi*		NC_007225	GenBank
Anthozoa	Hexacorallia	*Montipora cactus*		NC_006902	GenBank
Anthozoa	Hexacorallia	*Mussa angulosa*		NC_008163	GenBank
Anthozoa	Hexacorallia	*Nematostella* sp. JVK-2006		NC_008164	GenBank
Anthozoa	Hexacorallia	*Pavona clavus*		NC_008165	GenBank
Anthozoa	Hexacorallia	*Pocillopora damicornis*		NC_009797	GenBank
Anthozoa	Hexacorallia	*Pocillopora eydouxi*		NC_009798	GenBank
Anthozoa	Hexacorallia	*Polycyathus* sp.		NC_015642	GenBank
Anthozoa	Hexacorallia	*Porites okinawensis*		NC_015644	GenBank
Anthozoa	Hexacorallia	*Porites porites*		NC_008166	GenBank
Anthozoa	Hexacorallia	*Ricordea florida*		NC_008159	GenBank
Anthozoa	Hexacorallia	*Savalia savaglia*		NC_008827	GenBank
Anthozoa	Hexacorallia	*Savalia savaglia*		NC_008827	GenBank
Anthozoa	Hexacorallia	*Seriatopora caliendrum*		NC_010245	GenBank
Anthozoa	Hexacorallia	*Seriatopora hystrix*		NC_010244	GenBank
Anthozoa	Hexacorallia	*Siderastrea radians*		NC_008167	GenBank
Anthozoa	Hexacorallia	*Stylophora pistillata*		NC_011162	GenBank
Anthozoa	Octocorallia	*Acanella eburnean*		EF672731	GenBank
Anthozoa	Octocorallia	*Anthomastus* sp.	1171062	KM015352	This study
Anthozoa	Octocorallia	*Anthomastus* sp.	1081145	KM015353	This study
Anthozoa	Octocorallia	*Briareum asbestinum*		NC_008073	GenBank
Anthozoa	Octocorallia	*Calicogorgia granulosa*		GU047880	GenBank
Anthozoa	Octocorallia	*Corallium japonicum*		AB595189	GenBank
Anthozoa	Octocorallia	*Dendronephthya castanea*		GU047877	GenBank
Anthozoa	Octocorallia	*Dendronephthya gigantea*		NC_013573	GenBank
Anthozoa	Octocorallia	*Dendronephthya mollis*		HQ694725	GenBank
Anthozoa	Octocorallia	*Dendronephthya putteri*		HQ694726	GenBank
Anthozoa	Octocorallia	*Dendronephthya suensoni*		GU047878	GenBank
Anthozoa	Octocorallia	*Echinogorgia complexa*		HQ694727	GenBank
Anthozoa	Octocorallia	*Euplexaura crassa*		HQ694728	GenBank
Anthozoa	Octocorallia	*Hemicorallium imperiale*	1072448	KC782352	Figueroa and Baco (2014)
Anthozoa	Octocorallia	*Hemicorallium imperiale*	1072449	KC782355	Figueroa and Baco (2014)
Anthozoa	Octocorallia	*Hemicorallium laauense*		KC782348	Figueroa and Baco (2014)
Anthozoa	Octocorallia	*Keratoisinidae* sp.		EF622534	GenBank
Anthozoa	Octocorallia	*Narella hawaiinensis*	1072109	KM015351	This study
Anthozoa	Octocorallia	*Paragorgia* sp.	1075769	KC782349	Figueroa and Baco (2014)
Anthozoa	Octocorallia	*Paragorgia* sp.	1075761	KC782350	Figueroa and Baco (2014)
Anthozoa	Octocorallia	*Paragorgia* sp.	1072362	KC782351	Figueroa and Baco (2014)
Anthozoa	Octocorallia	*Paragorgia* sp.	1072339	KC782354	Figueroa and Baco (2014)
Anthozoa	Octocorallia	*Paragorgia* sp.	1075741	KC782356	Figueroa and Baco (2014)
Anthozoa	Octocorallia	*Paraminabea aldersladei*		JX508792	GenBank
Anthozoa	Octocorallia	*Pleurocorallium kishinouyei*	1072441	KC782353	Figueroa and Baco (2014)
Anthozoa	Octocorallia	*Pleurocorallium konojoi*		NC015406	GenBank
Anthozoa	Octocorallia	*Pleurocorallium secundum*		KC782347	Figueroa and Baco (2014)
Anthozoa	Octocorallia	*Pseudopterogorgia bipinnata*		NC_008157	GenBank
Anthozoa	Octocorallia	*Renilla muelleri*		JX023273.1	GenBank
Anthozoa	Octocorallia	*Sarcophyton glaucum*		AF063191	GenBank
Anthozoa	Octocorallia	*Scleronephthya gracillimum*		GU047879	GenBank
Anthozoa	Octocorallia	*Sibogagorgia cauliflora*	1122229	KM015354	This study
Anthozoa	Octocorallia	*Sinularia peculiaris*		NC_018379	GenBank
Anthozoa	Octocorallia	*Stylatula elongate*		NC_018380	GenBank
Medusozoa	Hydrozoa	*Clava multicornis*		NC_016465	GenBank
Medusozoa	Hydrozoa	*Craspedacusta sowerbyi*		JN593332	GenBank
Medusozoa	Hydrozoa	*Craspedacusta sowerbyi*		NC_018537	GenBank
Medusozoa	Hydrozoa	*Cubaia aphrodite*		NC_016467	GenBank
Medusozoa	Hydrozoa	*Hydra magnipapillata*		NC_008411	GenBank
Medusozoa	Hydrozoa	*Hydra oligactis*		NC_008071	GenBank
Medusozoa	Hydrozoa	*Laomedea flexuosa*		NC_016463	GenBank
Medusozoa	Scyphozoa	*Aurelia aurita*		HQ694729	GenBank
Medusozoa	Scyphozoa	*Aurelia aurita*		NC_008446	GenBank
Medusozoa	Scyphozoa	*Cassiopea frondosa*		NC_016466	GenBank
Medusozoa	Scyphozoa	*Chrysaora quinquecirrha*		HQ694730	GenBank
Porifera	Demospongiae	*Agelas schmidti*		NC_010213	GenBank
Porifera	Demospongiae	*Amphimedon compressa*		NC_010201	GenBank
Porifera	Demospongiae	*Aplysina fulva*		NC_010203	GenBank
Porifera	Demospongiae	*Igernella notabilis*		NC_010216	GenBank
Porifera	Demospongiae	*Oscarella carmela*		NC_009090	GenBank

### DNA Extraction, PCR, Sequencing and Assembly

Total genomic DNA was extracted from each specimen using Qiagen’s DNeasy Blood and Tissue Kit. Complete mitochondrial genomes of each specimen were obtained using a series of overlapping polymerase chain reactions (PCRs) using previously published primers sets (Park et al. 2012; Figueroa and Baco 2014) (table 2). The following thermocycling conditions were used: 96 °C for 2 min, 35 cycles at 94 °C for 1 min, 48 °C for 1 min, 72 °C for 1 min, and a final step at 72 °C for 5 min. The PCR fragments were sent for sequencing at the University of Washington High Throughput Genomics Center for both the forward and reverse strands.

**Table 2:** Primers Used for this Study

Forward	Primer	Reverse	Primer	Start	End	Size (bp)	Overlap
1F	ATGAACAAATATCTTACACG	1R	ATAARTGCTGRAATAAAAT	1	699	698	162
2F	ACAACATTTTTTGATCCT	2R	GCTAAACCCAAGAAATG	667	1,290	623	32
3F	ACAGGTTATAGTTATAATGA	3R	GTCTGCTGGCACTTAGTTAG	1,223	1,860	637	67
4F	CTGGTCGAAGATGCGTAGTA	4R	TGTGCTAACACTGGGTTAGA	1,743	2,500	757	117
5F	TATGCGCTACATTCTCCTAT	5R	CACACTTCATAGCTAATCAT	2405	3,128	723	95
ssRNA-F1	CTGCGTTTAATACGTACTTGGC	6R	YACTGCATCTAAACCTATCA	2,680	3,591	911	448
7F	ATTCTAGGAATGGGCTGC	7R	GACATTTGTCCCCAAGGTAA	3,509	4,126	617	82
8F	ATATTTTAAGAGACGTTAAT	8R	CTCTACTGGATTAGCCCCTA	3,964	4,726	762	162
9F^a^	ATCCTTTAGTAACTCCTG	msh2806R	TAACTCAGCTTGAGAGTATGC	4,501	5,088	587	225
9F^a^	ATCCTTTAGTAACTCCTG	msh3101R	GATATCACATAAGATAATTCCG	4,527	5,354	827	561
10F	YTRCTTCAAATGGGGTTTCC	mutS-3458R	TSGAGCAAAAGCCACTCC	5,268	5,731	463	86
10F	YTRCTTCAAATGGGGTTTCC	mutS-6088R^a^	TGTGATAGGGTTGAGAAG	5,268	5,900	632	463
10F	YTRCTTCAAATGGGGTTTCC	10R	AGAATTGTAACACTCGGG	5,268	5,939	671	632
mutS-F5	ATTTAATTAAGAATCTCCAACTTCC	mutS-6979^a^	TATTAATGGGTGTCGGAG	5,932	6,937	1,005	7
mutS-6818F^a^	CTAAGCTATTTTTWCCCC	mutS-R2	TCTAAAGACTCATTAAGATAAACCC	6,918	7,875	957	19
13R	CTGTTTCCAAGCCTACTT	13F	CTATTTTAGGYTGGAAGAGA	7,861	8,623	762	14
14R	TTTCCTCTTGAGACAGTA	14F	ACTGGTGTAGTAAGACTA	8,516	9,219	703	107
octo2-H	CGATAAGAACTCTCCGACAATA	15F	CAACTGAATGGCCGCGGTAA	9,134	9,601	467	85
octo1-L	AGACCCTATCGAGCTTTACTGG	nd2-R1	GTTCAAGCTCTCCTGTGGAGCC	9,343	10,394	1051	258
nd2-1418R	ACATCGGGAGCCCACATA	16S-647F	ACACAGCTCGGTTTCTATCTACAA	9,772	10,552	780	622
16R	GCACGATAGATAATAGCGCA	16F	TGGTGACACAGCTCGGTT	9,791	10,590	799	761
17R	ATATTTGTTATTACTAAAGG	17F	ATTRTTATTTAAAGTATCTG	10,527	11,153	626	63
18R	TCCCAACCRATAAATARTTG	18F	GTTTTTAACTAARTGGTATR	11,043	11,709	666	110
19R	GCATGAATRATTGAGCCTGC	19F	ATTCTACAAGTTATATGAGA	11,605	12,323	718	104
20R	TATCATTAATGCATAATTAA	20F	AGTTTATATCAYYTACTAAC	12,299	13,051	752	24
21R	AACATTAAACTGAGCCGACT	21F	TGTCTCTTATCGTACTATAG	13,005	13,653	648	46
22R	TTTTATTATTAGTTAACCTTCATC	nad4-F3	TTTTATTATTAGTTAACCTTCATC	13,514	14,179	665	139
22R	GTACTAGTWGAAAAAGCAGC	nd4-13343F^a^	AATAGGTTGGTTTGAGGG	13,514	14,300	786	665
co3bam567F	GCTGCTAGTTGGTATTGGCAT	23F	ATGGTRTTTACTTTAGCTAA	14,264	14,787	523	36
23R	GCTGCTAGTTGGTATTGGCA	23F	ATGGTRTTTACTTTAGCTAA	14,274	14,835	561	513
24R	TATCACCCTTATCATYTAGT	24F	CTAAGARCCCCACCARTAAA	14,772	15,508	736	63
25R	TCWACAGCTAAYAAGGGAAC	25F	TGAAAATATARTACTGAGCC	15,468	16,063	595	40
siro-cox2-F1	AGGCCCACTCTGTATATTTC	atp6-R2	ATGTAGATTTAGAGTATCATTAATRTA	15,588	16,291	703	475
26R	CATTAGSTATTAAAATGGAT	26F	GTAAATACRTAGGGAAATAG	15,524	16,597	1,073	767
cox2-16530F^a^	CCCCTAAAGATCACCACA	nd42599F	GCCATTATGGTTAACTATTAC	16,582	17,397	815	15
27F	GAGTGATTAGCGCCACATAA	27R	GGAGCCTATATCCTTGRGAT	16,681	17,468	787	716
REVNRnd6^a^	ATCGTTAGCGGGACATTATCAATT	coII-8068F	CCATAACAGGACTAGCAGCATC	17,207	17,995	788	261
nd6-F	TCCTTAGGAATAGTTGGAGCTAG	nd3-2126R	CACATTCATAGACCGACACTT	17,935	18,600	665	60
siro-nad6-R1	ATTGCCCCTATGTTAGTTCTAG	28R	CCAATCATTACTGGCATTAC	18,304	233	982	296
nd6-F REV	CTAGCTCCAACTATTCCTAAGGA	New NCR2R	ATGATCATCTCCTAACATACTACC	18,774	162	585	162
9F^b^	ATCCTTTAGTAACTCCTG	COII-8068F	CCATAACAGGACTAGCAGCATC	4,531	5,123	593	—
msh2806R^b^	TAACTCAGCTTGAGAGTATGC	RevNrND6	ATCGTTAGCGGGACATTATCAATT	17,209	18,037	829	—

Note.—Unless otherwise noted, sequence numbers are based on mt genomes with *konojoi* gene arrangement, starting with *cox1*.

All primers are from previous research (Brugler and France 2008; Uda et al. 2011; Park et al. 2012, Figueroa and Baco 2014).

^a^Primer pairs used for mt genomes with *konojoi* arrangement only.

^b^Primer pairs used for mt genomes with *japonicum* arrangement only.

The overlapping PCR fragments were assembled using the software CLC Main Workbench 6.7.1 (CLC Bio, Aarhus, Denmark). Sequence quality was assessed by base quality scores and by visually inspecting each chromatogram. Annotation of each mitochondrial genome was done by alignment to all octocoral genomes available in GenBank (table 1) with the aid of the software CLC Main Workbench. The mt genomes were scanned for transfer ribonucleic acids (tRNAs) using the program tRNA scan-SE by Lowe and Eddy (1997).

### Substitution Saturation Analysis

A hierarchical substitution saturation analysis was performed at varying taxonomic levels to determine the potential phylogenetic signal contained in the nucleotide sequences of the mitochondrial genomes. There were three steps to this analysis. First, transitions and transversions were plotted against divergence based on general time reversible (GTR) distances (a GTR model was selected as the best fitting evolutionary model by our phylogenetic analysis, see next section). Second, the statistical tests presented by Steel et al. (1993) were used to determine how many sequences in each data set were phylogenetically informative. And third, saturation indices were calculated using the method by Xia et al. (2003) to determine whether the genomes have experienced substitution saturation. All three steps were carried out with the software package DAMBE (Xia and Xie 2001). This analysis was repeated for five groupings of the overall data set: Octocorallia only, Hexacorallia only, Anthozoa (Octocorallia + Hexacorallia), Cnidaria (Anthozoa + Medusozoa), and Cnidaria + Porifera.

### Phylogenetic Analysis

In addition to the four specimens used in this study, 82 mitochondrial genomes were obtained from GenBank and included in the phylogenetic analysis: 30 Octocorallia, 33 Hexacorallia, 7 Hydrozoa, 4 Scyphozoa, and 5 Porifera (table 1). The sequences for each gene and ribosomal RNA were aligned with MUSCLE (Edgar 2004) and then sequentially concatenated. The alignment was visually inspected for optimality. All phylogenetic analyses were performed with MEGA v5.05 (Tamura et al. 2011) using maximum-likelihood (ML) methods with bootstrap values from 10,000 replicates. A GTR model with gamma distribution and invariant sites (GTR + G + I) was selected by MEGA v5.05 as the best fitting model of molecular evolution based on the Akaike Information Criterion. Bayesian analyses were performed with MrBayes 3.1 (Ronquist and Huelsenbeck 2003) using a GTR + G + I model of evolution as selected by MrModeltest 2.2 (Nylander 2004). The chains were carried out for 5,000,000 generations, sampling every 500th generation. After inspecting the trace files generated by the Bayesian Markov chain Monte Carlo (MCMC) runs, we determined that the initial 25% (2,500) of sampled generations would be omitted.

For the phylogenetic reconstruction of Octocorallia, all 14 protein-coding genes, including the *mutS* gene, and 2 RNAs were used. For the phylogenetic reconstructions of both Anthozoa and Cnidaria, only 13 protein-coding genes were used. This is because the *mutS* gene is only found in octocorals and therefore could not be used in phylogenies above this taxonomic level. The two RNAs were also not included because they varied so much among higher taxa that homologous regions could not be accurately aligned.

#### Testing Phylogenetic Robustness

Because our inferences on gene order evolution within the Octocorallia rely heavily on their phylogeny, additional analyses were performed on this group to test the robustness of the reconstructed phylogeny. Starting with the alignment, the visual inspection for optimality was compared with alignment optimization using the software GBLOCKS 0.91b (Castresana 2000) using default settings with “Allowed GAP positions” set to “All.” The ML and Bayesian analyses, as described above, were repeated with the alignment selected by GBLOCKS. Because multiple coding genes were used, a partitioned phylogenetic analysis was also performed using PartitionFinder v1.1.1 (Lanfear et al. 2014) and RAxML v8.0.0 (Stamatakis 2014). To find the optimal ML tree with RAxML, 20 independent searches were performed with 1,000 bootstrap replicates. Data blocks were defined by each gene and codon position for the 14 protein-coding genes. Codon positions were not used for the two RNAs. Finally, four additional, independent Bayesian analyses were run using MrBayes 3.1 (Ronquist and Huelsenbeck 2003) with a GTR + G + I model of evolution as selected by MrModeltest 2.2 (Nylander 2004). The chains were carried out for 1,000,000 generations, sampling every 100th generation. The software AWTY (Wilgenbusch et al. 2004) was then used to test for convergence of the MCMC runs.

## Results

### Mitochondrial Genomes

Four new octocoral mitochondrial genomes were obtained. All four have similar lengths, from shortest to longest: 18,716 bp (*Anthomastus* sp. USNM# 1171062), 18,838 bp (*N. hawaiinensis* USNM# 1072109), 18,913 bp (*Anthomastus* sp. USNM# 1081145), and 19,044 bp (*S. cauliflora* USNM# 1122229). All 4 mt genomes contain 14 protein-coding genes (atp6, atp8, cox 1–3, cob, nad 1–6, nad4L, and mutS), 2 ribosomal RNAs (12s and 16s), and 1 transfer RNA. The A + T content in all four mt genomes is similar, ranging from 62.2% to 63.3%. The nucleotide lengths of all genes are similar for all four species.

**Fig. 1.—:**
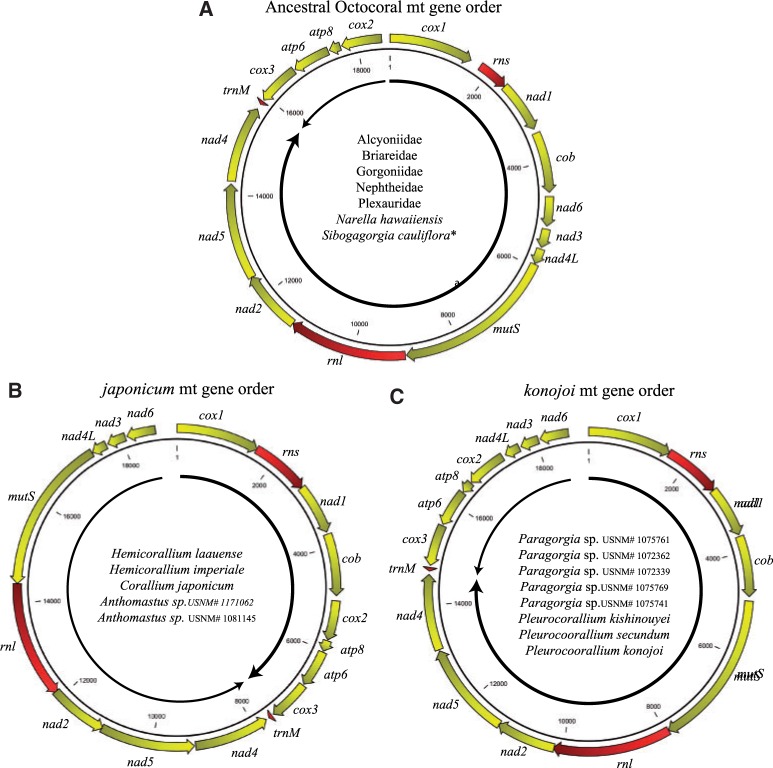
Mitochondrial gene arrangement based on Medina et al. (2006), Brugler and France (2008), Park et al. (2012), Uda et al. (2011), Figueroa and Baco (2014), and this study. Arrows show direction of replication. Thicker line shows heavy strand, thinner line shows light strand. (*A*) Presumed octocoral ancestral mt gene arrangement; (*B*) *japonicum* mt gene arrangement; and (*C*) *konojoi* mt gene arrangement. Taxa that have been shown to have these arrangements are listed within each arrangement. *Although *Sibogagorgia cauliflora* has the presumed ancestral gene order, it is not because it was conserved in this lineage but rather it reversed back from a different arrangement to this ancestral state, as explained in the text.

**Fig. 2.—:**
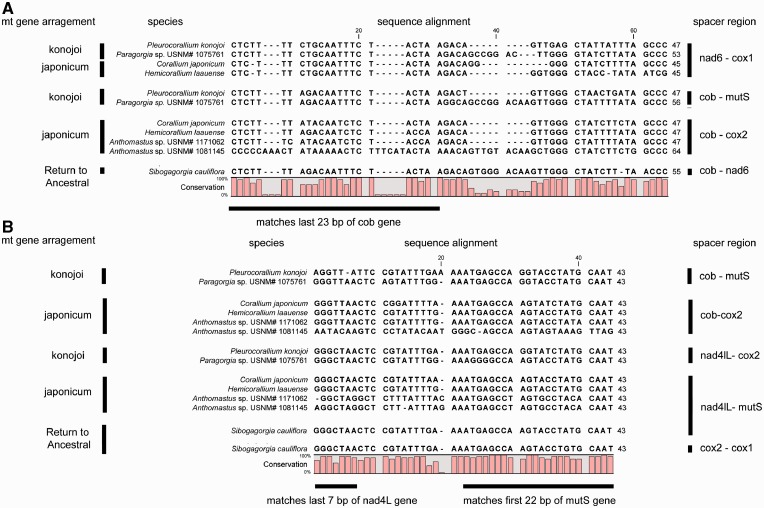
Alignment of inverted repeat sequences present in all the *Corallium*, *Paragorgia*, *Anthomastus*, and *Sibogagorgia* mitochondrial genomes. These were first identified by Uda et al. (2011) and they occur in the intergenic spacers where gene inversions took place leading to the *japonicum* and *konojoi* mt gene arrangement. Panel A corresponds to spacer *a* and Panel B to spacer b shown in figures 5 and 7.

Two gene arrangements were observed (fig. 1), both species of *Anthomastus* have the same arrangement as that discovered by Uda et al. (2011) in *Corallium japonicum*, further referred to as the “*japonicum*” arrangement; while *N. hawaiinensis* and *S. cauliflora* both have what is assumed to be the ancestral arrangement in octocorals (McFadden et al. 2006; Uda et al. 2011; Brockman and McFadden 2012) (fig. 1). In all 4 mitochondrial genomes, 7 of the genes either overlap or do not have a spacer between them, with the rest separated by a total of 12 intergenic spacers, ranging in size from 14 to 396 bp. Within the spacers, the two *Anthomastus* mt genomes and the *Sibogagorgia* mt genome have one pair of an inverted repeat sequence (fig. 2), identified previously in the mitochondrial genomes of *C. japonicum* and *Pleurocorallium konojoi* (Uda et al. 2011). In *Anthomastus*, these inverted repeat sequences are found in the intergenic regions between *cob* and *cox2* genes and *mutS* and *nad4L* genes; while in *Sibogagorgia*, they are found in the intergenic regions between *cob* and *nad6* genes, *nad4L* and *mutS* genes, and *cox1* and *cox2* genes (fig. 2).

### Substitution Saturation Analysis

**Fig. 3.—:**
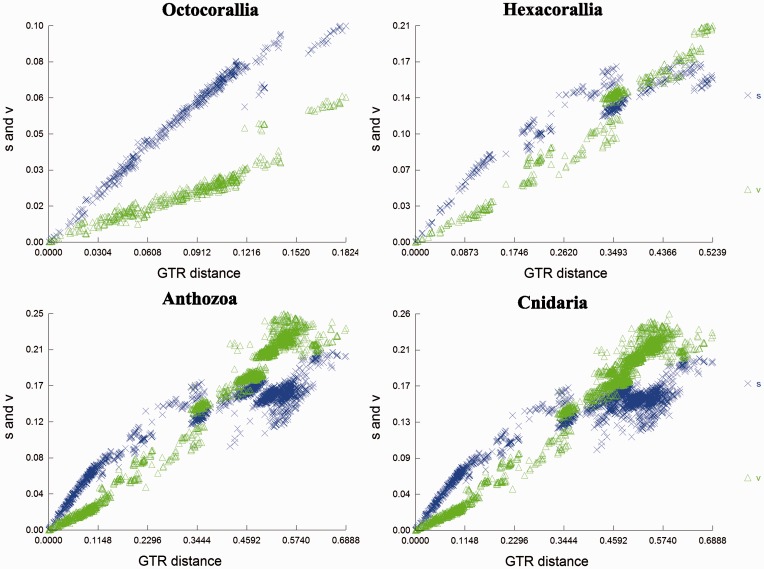
Transitions (s) and transversions (v) compared with GTR distance for four data sets: within the subclass Octocorallia, within the subclass Hexacorallia, within the class Anthozoa, and within the phylum Cnidaria (Anthozoa + Medusozoa).

Plots of transitions and transversions versus divergence based on GTR distances (fig. 3) show a linear relationship for the Octocorallia, with transitions always greater than transversions. For the Hexacorallia, the relationship between transversions and divergence is linear, while the relationship between transitions and divergence starts out linear and then levels off at higher divergences. Also, at these higher divergences transversions begin to surpass transitions. For the Anthozoa (Hexacorallia + Octocorallia) and the Cnidaria (Hexacorallia +Octocorallia + Medusozoa), the relationship between transitions and transversions versus divergence is comparable with that described above for the Hexacorallia. One exception is that in the Cnidaria transversions start to level off at higher divergences and transitions begin to lose their linear relationship and are surpassed by transversions at a lower divergence. When the Porifera are added to the Cnidaria data set (not shown in figure), the relationships are similar to that of the Cnidaria; however, the linearity of the relationship for both transitions and transversions is lost at even lower divergence levels.

**Fig. 4.—:**
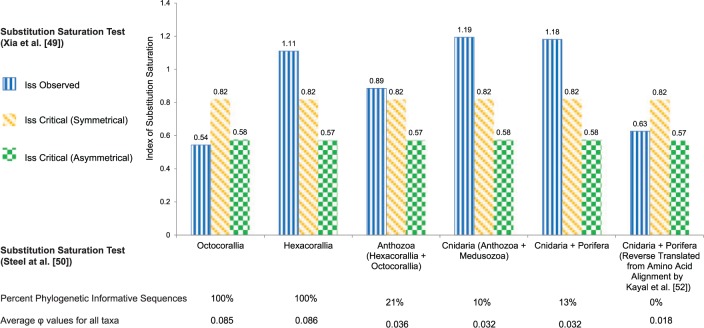
Substitution saturation tests for six data sets as implemented by DAMBE, based on Xia et al. (2003) and Steel et al. (1993). Graph shows Iss observed and Iss critical for both symmetrical and asymmetrical tree. If Iss observed is higher than Iss critical, then it means that the sequences have high substitution saturation and will fail to recover the phylogenetic signal. All differences are significant. The *P* value for all comparisons is 0.0000 except for the Iss observed versus Iss asymmetrical in Octocorallia, were the *P* value is 0.029. Below the graph is the average φ value from Steel et al.’s test for each data set. A value of less than 0.04 is considered to lack a phylogenetic signal (Xia and Lemey 2009). Sequences above this threshold are considered phylogenetically informative and are shown as a percentage.

The results for the substitution saturation index defined by Xia et al. (2003) are shown in figure 4. The test, as implemented by DAMBE, calculates a critical index for a symmetrical and an asymmetrical tree and compares it with the observed index (Iss). If the Iss observed value is higher than the Iss critical values, then the sequences will fail to recover the true phylogenetic relationships. The index shows that for the Octocorallia the observed Iss is lower than either of the critical values. For all the remaining data sets Hexacorallia, Anthozoa, Cnidaria, and Cnidaria + Porifera, the Iss observed is higher than either of the critical values. The statistical test by Steel et al. (1993) as implemented in DAMBE gives each sequence a φ score from 0 to 1 based on how phylogenetically informative that sequence is relative to what can be expected by chance. A score below 0.04 is considered as lacking phylogenetic information (Xia and Lemey 2009). These test results are summarized in figure 4 and show that for Octocorallia and Hexacorallia all the sequences are phylogenetically informative. For the Anthozoa only 21% of the sequences are phylogenetically informative, for the Cnidaria only 10%, and for the Cnidaria + Porifera only 13%.

One way to deal with possible substitution saturation is to translate nuclear sequences to amino acid sequences, and then reverse translate them back to nucleotide sequences using a universal code. This effectively gets rid of synonymous substitutions and it is a method used by Park et al (2012) for their study on cnidarian divergence times using whole mitochondrial genomes. Another option is to reconstruct phylogenies using the amino acid sequences themselves after translating nuclear sequences. This method was utilized by Kayal et al. (2012) for reconstructing the phylogeny of the Cnidaria. As part of our analysis, we used the amino acid alignment from Kayal et al. (2012) and reverse translated the alignment following the same procedures as Park et al. (2012). We performed both saturation tests on this data set. There was a marked improvement with respect to Iss scores for the Xia et al. (2003) test compared with our Cnidaria and Cnidaria + Porifera data set (fig. 4). But, it only passes the test if the tree is symmetrical while still failing the test if the resulting tree is asymmetrical. Although the Xia et al. (2003) substitution saturation test did show some improvement, the test by Steel et al (1993) showed that all the sequences in this new data set were lacking phylogenetic information and therefore any tree recovered could statistically be due to chance.

### Octocorallia Phylogenetic Analysis

**Fig. 5.—:**
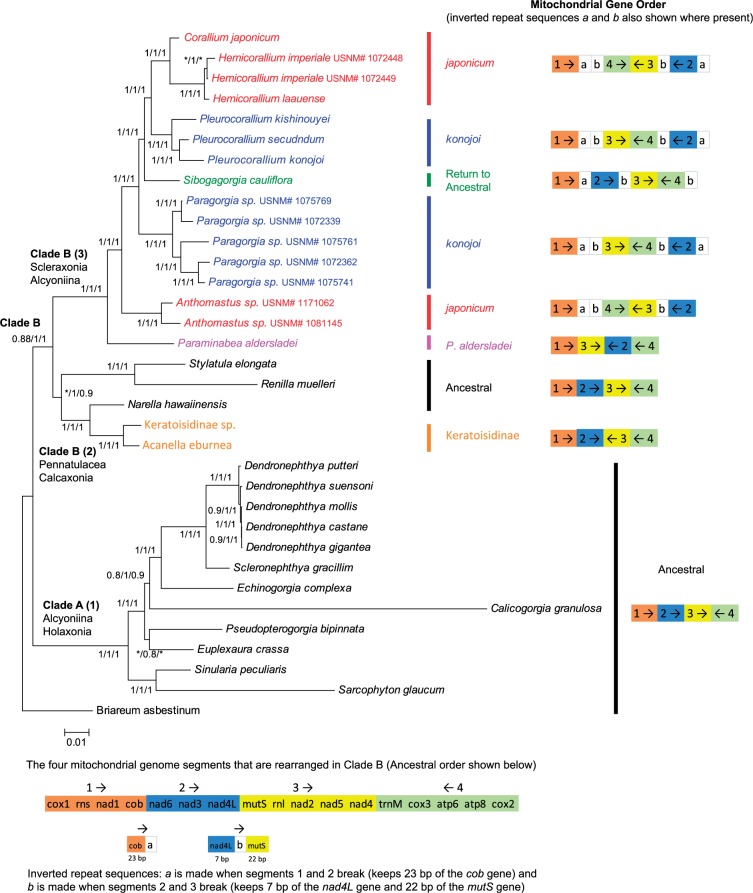
Octocoral phylogenetic tree inferred by ML, based on all mitochondrial protein-coding genes and RNAs. The tree is drawn to scale, with branch lengths measured in the number of substitutions per site. Tree topology inferred by Bayesian methods is identical except for *Hemi*c*orallium imperiale* USNM# 1072449 branches with *Hemi*c*orallium imperiale* USNM# 1072448 in the Bayesian topology and with *Hemi*c*orallium laauense* in the ML topology. Branch values correspond to bootstrap support for ML (first) and Bayesian posterior probabilities (second) for the nonpartitioned data. The third branch value corresponds to bootstrap support for ML as determined by RAxML with the partitioned data. *Support values less than 0.70. Clade numbers are labeled to correspond to the clade designations in McFadden et al. (2006). Coloring corresponds to the different mitochondrial gene orders as shown to the right of the phylogeny. The corresponding genes in each numbered box are given in the bottom panel of the diagram.

A total of 34 octocoral mitochondrial genomes were used in the octocoral phylogenetic analysis, using all 14 protein-coding sequences and the 2 ribosomal RNAs. Our original alignment was very similar to the alignment selected by GBLOCKS 0.91b where 98% of the original 18,398 bp were retained. Phylogenetic analyses were performed on both alignments and they yielded identical results. The same tree topology was obtained with both ML and Bayesian methods (five independent Bayesian analyses) and both methods resulted in well-supported branches (fig. 5). Analyses using the software AWTY showed convergence of all MCMC runs. All runs yielded identical topology and branch support. Both analyses were performed unrooted; once the tree was obtained, it was then redrawn with *Briareum asbestinium* as the root because this species is considered to be basal in the Octocorallia (McFadden et al. 2006; Brockman and McFadden 2012; Park et al. 2012). PartitionFinder v1.1.1 (Lanfear et al. 2014) divided the data into six partitions. The partitioned phylogenetic analysis performed with RAxML included 20 independent searches for the optimum ML tree with 1,000 bootstrap replicates. This also yielded the same phylogenetic tree with similar support for all branches with only one exception. Our original tree shows that the species *Euplaxaura crassa* and *Pseudopterogogia bipinnata* are sister taxa, while the partitioned analysis collapses this clade.

The tree shows two main clades, which we will refer to as Clade A and Clade B. Clade A contains members of the suborders Alcyoniina and Holaxonia and includes the same groups of taxa which fall into Clade 1 of McFadden et al. (2006). All members of the Clade A have the presumed ancestral octocoral mitochondrial gene arrangement. Clade B contains the other members of the order Pennatulacea and of the suborders Alcyoniina, Calcaxonia, and Scleraxonia. All four alternate gene arrangements are found in the members of Clade B. This clade splits into two clear subclades, Clade B(2), containing the Pennatulacea and Calcaxonia, and corresponding to Clade 2 of McFadden et al. (2006), and the second, Clade B(3) containing the Scleraxonia and the two Alcyoniina *Paraminabea* and *Anthomoastus*, corresponding to Clade 3 of McFadden et al. (2006). In Clade B(3), *Paraminabea* branches out first, then *Anthomastus* forms a sister branch with Paragorgiidae and Coralliidae. The Paragorgiidae is a paraphyletic taxon, because *Sibogagorgia* does not group with the *Paragorgia*, but rather forms a sister branch to the Coralliidae. The Coralliidae have two main branches, one leading to *Corallium* and *Hemicorallium*, all with the *japonicum* mitochondrial gene arrangement, while the other leading to *Pleurocorallium*, which have the *konojoi* mitochondrial gene arrangement.

### Anthozoa and Cnidaria Phylogenetic Analysis

**Fig. 6.—:**
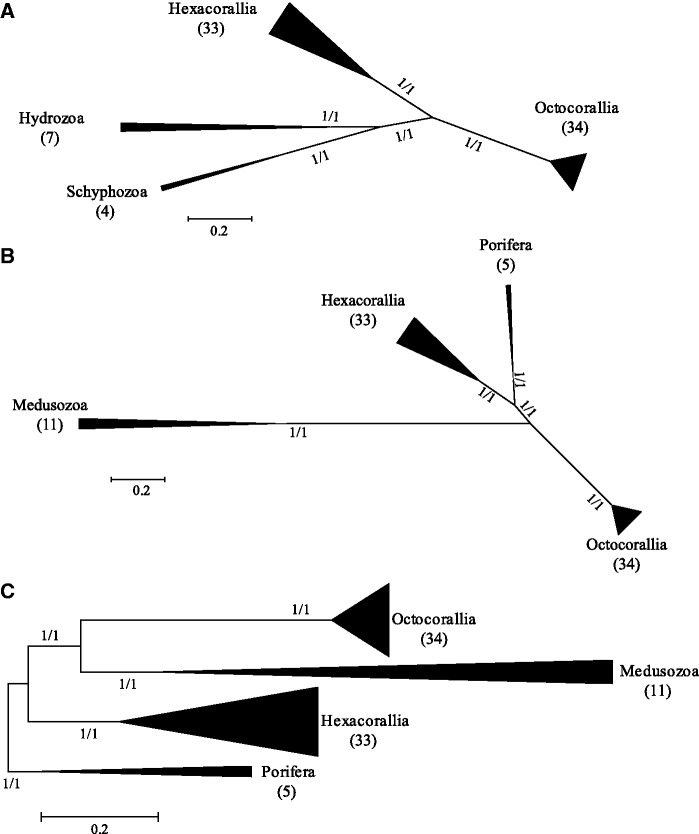
Phylogenetic trees for the Anthozoa (*A*, unrooted) and Cnidaria + Porifera (*B*, unrooted and *C*, rooted by the Porifera) inferred by ML, based on all mitochondrial protein-coding genes, excluding RNAs and *mutS*. The tree is drawn to scale, with branch lengths measured in the number of substitutions per site. Tree topology inferred by Bayesian methods is identical. Branch values correspond to bootstrap support for ML (first) and Bayesian posterior probabilities (second). The number of taxa in each branch is shown in parenthesis.

A total of 78 mitochondrial genomes were used for the phylogenetic reconstruction of the Anthozoa, 67 Anthozoa (34 Octocorallia and 33 Hexacorallia), and 11 Medusozoa (7 Hydrozoa and 4 Schyphozoa). Unlike the Octocorallia phylogenetic analysis, only 13 protein-coding genes were concatenated and aligned. The Octocorallian *mutS* gene was excluded as it is not present in any other taxa and the two RNAs were also excluded due to high levels of variation in large gaps in alignments above the subclass level, making it difficult for homologous regions to be aligned. Both ML and Bayesian methods resulted in similar tree topology with well-supported branches (fig. 6*A*). The medusozoans were included in this analysis as an outgroup for the Anthozoa and they form a distinct clade that divides into two branches, one containing the Hydrozoa and the other the Schyphozoa. There was no support for an Anthozoan clade. Instead, the Octocorallia and the Hexacorallia branched independently. The internal branching of the Octocorallia is similar to that of the previous analysis but some resolution has been lost including the collapse of some branches (tree not shown).

The same mt genomes used in the Anthozoa analysis were used for the Cnidarian analysis with the addition of five Porifera as the outgroup. The *mutS* gene and the two RNAs were also omitted in this analysis. The phylogenetic reconstruction shows four distinct and well-supported clades: 1) The Porifera, 2) the Medusozoans, 3) the Hexacorallia, and 4) the Octocorallia (fig. 6*B*). When the tree is redrawn, using the Porifera as the root for the Cnidaria, the Hexacorallia form the first derived branch for this group, while the Medusozoans and Octocorallia form a second branch (fig. 6*C*).

## Discussion

### Phylogeny of the Octocorallia

Our phylogenetic reconstruction supports two major clades within the Octocorallia (fig. 5). One includes the Alcyoniina and Holaxonia, while the other divides into two branches, one composed of Pennatulacea and Calcaxonia, and the second with *Anthomastus*, Paragorgiidae, and Coralliidae. This largely agrees with the phylogeny proposed by McFadden et al. (2006) except that in their study the basal relationships between these three clades remain inconclusive, while here they are more supported. Their phylogenetic analysis is based on two mitochondrial genes, *nad2* and *mutS*. When using maximum parsimony and Bayesian methods, their phylogeny show Clades A(1) Alcyoniina and Holaxonia and B(3) *Anthomastus*–Paragorgiidae–Coralliidae as sister clades, while their reconstruction using ML shows Clades B(2) Pennatulacea and Calcaxonia and B(3) *Anthomastus*–Paragorgiidae–Coralliidae as sister branches. Our phylogenetic reconstruction supports the latter. Using the entire mitochondrial genome provides robust support for an independent Clade A(1) Alcyoniina–Holaxonia. Clades B(2) Pennatulacea–Calcaxonia and B(3) *Anthomastus*–Paragorgiidae–Coralliidae have strong support as sister clades in the Bayesian analysis. Our ML analysis recovers the same relationships, but in this case the support for a sister relationship between Clades B(2) and B(3) is weaker.

The phylogenetic relationships within Clades A(1) Alcyoniina–Holaxonia and B(2) Pennatulacea–Calcaxonia are discussed at length by McFadden et al. (2006). The number of full mitochondrial genomes available for members of these two clades is limited, 12 for Clade A(1) and 5 for Clade B(2), when compared with the number of taxa used in McFadden et al. (2006) where there are 73 for the former and 24 for the latter. Therefore it will suffice to say that our limited data set for these two clades is congruent with that of McFadden et al. (2006) and we will defer further discussion to their study and the sequencing of further mt genomes. In the case of Clade B(3) *Anthomastus*–Paragorgiidae–Coralliidae, our study includes 16 members, while McFadden et al. (2006) only has 3. Our study shows that full mitochondrial genomes work well in resolving the phylogeny within this clade. *Paraminabea* is the basal member of this clade, followed by *Anthomastus*. In a recent taxonomic revision of *Anthomastus* based on morphology, it was suggested that this genus should be divided into at least three genera, *Anthomastus*, *Heteropolypus*, and *Pseudoanthomastus* (Molodtsova 2013). We support this taxonomic revision because *Anthomastus ritteri*, which has been revised by Molodstova (2013) as *Heteropolypus ritteri*, has the presumed octocoral ancestral gene order (Brockman and McFadden 2012), while our two morphospecies of what are presumably *Anthomastus* have a *japonicum* gene order. This genetic information supports at least two distinct lineages. Genetic support for the third lineage will have to wait until the full mitochondrial genomes of members of all three revised genera are sequenced.

After *Anthomastus*, the next branch in Clade B(3) is composed of *Paragorgia*. *Paragorgia* was erroneously thought to be a sister branch to the Coralliidae (Brockman and McFadden 2012; Uda et al. 2013; Figueroa and Baco 2014), but our results clearly show that the sister branch to the Coralliidae is *Sibogagorgia*. Both *Paragorgia* and *Sibogagorgia* currently belong to the family Paragorgiidae. Our phylogenetic analyses show that *Paragorgia* and *Sibogagorgia* are two independent lineages, making the Paragorgiidae a paraphyletic group. We propose that to fix this taxonomic inadequacy, the family Sibogagorgiidae, as suggested by Verseveldt (1942), should be resurrected for *Sibogagorgia*. *Sibogagorgia* was also found to be highly divergent in the analyses by Herrera et al (2010) based on mitochondrial genes. A less favorable alternative to make Paragorgiidae monophyletic would be to subsume the Coralliidae into the Paragorgiidae. The last branch in Clade B(3) has the members of the Coralliidae. The Coralliidae are clearly composed of three lineages, which support the recent split of *Corallium* into three genera, *Corallium*, *Hemicorallium*, and *Pleurocorallium* (Ardila et al. 2012; Figueroa and Baco 2014).

### Mitochondrial Gene Order: Evidence of Reversal to an Ancestral State

**Fig. 7.—:**
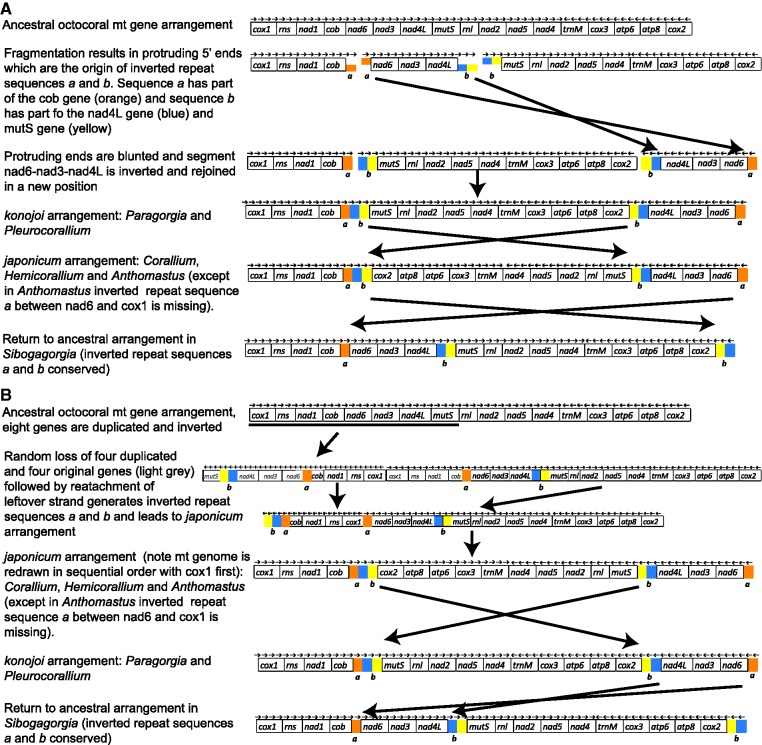
Theoretical origin of inverted repeat sequences *a* and *b* in Sibogagorgia. There are two possible scenarios. In Scenario *A*, the *konojoi* arrangement arises first, creating the two inverted repeat sequences; these are conserved in the subsequent evolution of the *japonicum* arrangement and in the return to an ancestral state in *Sibogagorgia*. In Scenario *B*, the *japonicum* arrangement arises first creating the inverted repeats; these are conserved in the subsequent evolution of the *japonicum* arrangement and in the return to an ancestral state in *Sibogagorgia*.

The four mitochondrial genomes of *N. hawaiinensis*, *S. cauliflora*, and the two morphospecies of *Anthomastus* have the same compositional elements as the mitochondrial genomes of all 29 species of octocorals that have been published to date (fig. 1). There are five different gene arrangements that have been identified in the Octocorallia (Beaton et al. 1998; Brugler and France 2008; Uda et al. 2011; Brockman and McFadden 2012; Park et al. 2012). Our study shows that *Anthomastus* has the same mitochondrial gene arrangement as the one discovered in *Paracorallium japonicum* by Uda et al. (2011) and also shared by at least three species of *Corallium* (Figueroa and Baco 2014). Both *N. hawaiinensis* and *S. cauliflora* have the presumed ancestral mitochondrial gene order. However, despite having the presumed ancestral gene order, the presence of a pair of inverted repeat sequences in the spacer regions of *S. cauliflora* suggest that this apparent ancestral mitochondrial gene arrangement was not conserved in this species but rather evolved back to its ancestral state after going through a rearrangement (fig. 7).

The inverted repeat sequences were first identified by Uda et al. (2011) in the mitochondrial genomes of both *P. japonicum* and *Corallium konojoi* and have since been identified in several other species of *Corallium* and *Paragorgia* (Figueroa and Baco 2014). The origin of these inverted repeat sequences are discussed in detail in Uda et al. (2011). The authors suggest two possible pathways for the origin of these inverted repeat sequences, one is going from a presumed ancestral mitochondrial gene arrangement to a *japonicum* arrangement, and the other is going from the presumed ancestral arrangement to a *konojoi* arrangement. Either pathways result in inversions leading to the inverted repeat sequences in the intergenic spacer regions that carry part of the gene to which they were previously adjacent. Uda et al. (2011) clearly show that the only way these inverted repeat spacer sequences can form is to go through either the *konojoi* or *japonicum* rearrangements. Therefore because these inverted repeat sequences are present in the mitochondrial genome of *S. cauliflora*, which has the presumed ancestral gene order, it suggests that the gene arrangement in this taxon is not an indication of a conserved ancestral state but rather that the gene order evolved back to the ancestral state from either a *konojoi* or a *japonicum* arrangement.

This is the first observation that shows that in the Octocorallia, mitochondrial gene arrangement is not only diverse but it can evolve back to an ancestral state. This has important implications for genetic studies that use gene boundaries to determine the type of mitochondrial gene arrangement present and then use that information for classification or phylogenetic purposes. This practice of testing gene boundaries has been referred to as “gene junction screening” (Brockman and McFadden 2012). If this were done with *S. cauliflora*, it would show that it has the ancestral gene arrangement and lead to the erroneous conclusion that *Sibogagorgia* is basal to *Paragorgia* and Coralliidae because those taxa have derived mitochondrial gene arrangements. But this is not the case, by analyzing the complete mitochondrial genome, including intergenic spacers, it is clear that the gene arrangement in *S. cauliflora* is also derived and has evolved back to an ancestral gene order. Therefore we recommend for future studies of gene rearrangements not to rely exclusively on gene junction screening as it will miss reversals to ancestral states.

### Evolution of Mitochondrial Gene Arrangements

**Fig. 8.—:**
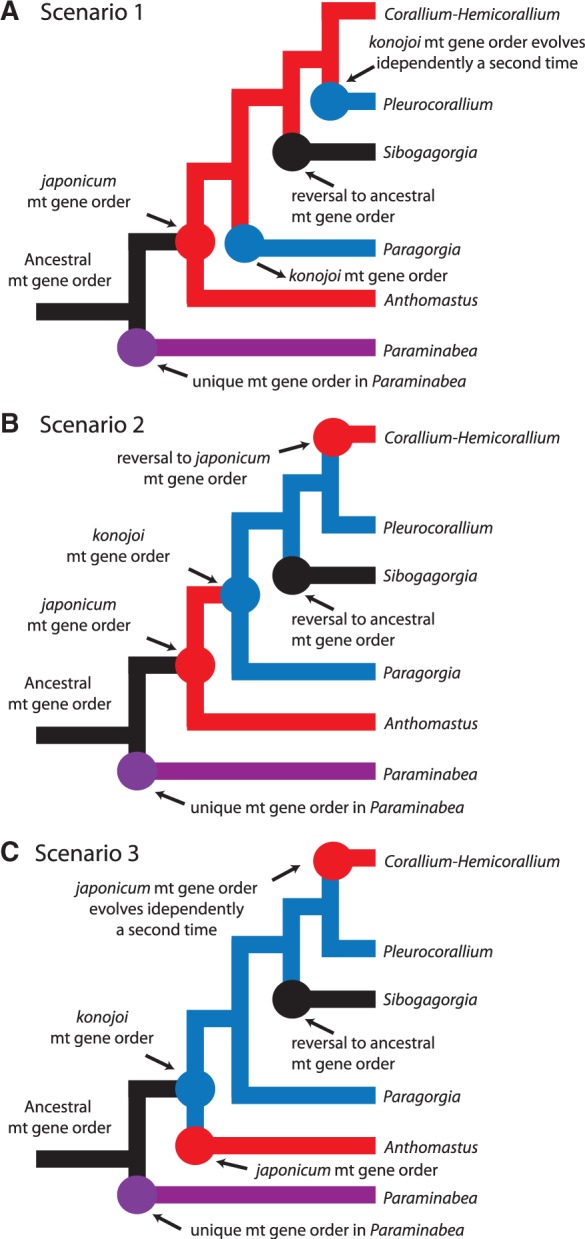
Three possible evolutionary pathways for the different mitochondrial gene orders found in Clade B(3) of the Octocorallia. All three scenarios are equally plausible in terms of the number of evolutionary steps needed. The tree is not drawn to scale. Arrows point to nodes where a particular gene order evolved. Branches are color coded for the gene orders, black for ancestral, purple for *Paraminabea*, red for *japonicum*, and blue for *konojoi*. Panel *A* shows Scenario 1 where the *japonicum* arrangement evolves first and it is conserved throughout going back to an ancestral state in *Sibogagorgia* and with the *konojoi* order evolving twice independently. Panel *B* shows Scenario 2 where the *japonicum* order also evolves first, but it is not conserved. Instead, the *konojoi* order evolves right and it is conserved afterwards, going back to an ancestral state in *Sibogagorgia* and with the *japonicum* order evolving independently a second time in the *Corallium–Hemicorallium* clade. Panel *C* shows Scenario 3 where the *konojoi* order evolves first and it is conserved throughout, going back to an ancestral order in *Sibogagorgia* and with the *japonicum* order evolving twice independently.

Our phylogenetic analysis shows that within the Clade B of the Octocorallia, mitochondrial gene order has changed at least six times. The first change occurs in the basal branch of this clade from the presumed ancestral gene order to the unique order shared by *Keratoisidinae* sp. and *Acanella eburnea* (fig. 5). The second change comes in the basal branch for Clade B(3) (fig. 5), going from the presumed ancestral gene order to the unique arrangement found in *Paraminabea aldersladei*. *Paraminabea aldersladei* is the sister branch to the rest of the members of Clade B(3) where presumably the ancestral gene order was maintained. From this point, there are three equally plausible scenarios for the evolution of the *japonicum* and *konojoi* gene order and the return to an ancestral state in *Sibogagorgia* (fig. 8).

In the first scenario, the third change occurs from the presumed ancestral gene order to the *japonicum* gene order (fig.8*A* and *B*) found in the two morphospecies of *Anthomastus*. The *japonicum* gene order is maintained and conserved through to the *Corallium* and *Hemicorallium* clade, while the *konojoi* gene order arises independently twice, once in *Paragorgia* and a second time in the *Pleurocorallium* (fig. 8*A*). In *Sibogagorgia*, it returns to an ancestral order from a *japonicum* arrangement (fig. 8*A*). In the second scenario, the *japonicum* gene order also evolves first from the presumed ancestral gene order, but then is only conserved in the *Anthomastus* clade, while the *konojoi* emerges as ancestral to the remaining branches (fig. 8*B*) and is therefore conserved in *Paragorgia* and *Pleurocorallium*. In this scenario in the *Corallium*–*Hemicorallium* clade, the gene order reverses to the *japonicum* arrangement and *Sibogagorgia* returns to the ancestral gene order from a *konojoi* arrangement (fig. 8*B*).

In the third scenario, the *konojoi* gene order evolves first (fig. 8*C*). The *konojoi* order is maintained throughout the main branch and conserved through to *Pleurocorallium*. In this case, the *japonicum* arrangement evolves independently, once in the *Anthomastus* and a second time in the *Corallium*–*Hemicorallium* clade and *Sibogagorgia* goes back to an ancestral state from a *konojoi* arrangement.

All three of these possible scenarios have the same number of evolutionary steps and in all three, one of the gene orders, *japonicum* or *konojoi*, had to evolve twice. Previous studies have also tried to determine the sequence of evolutionary events leading to these gene arrangements in Clade B (Uda et al. 2011; Brockman and McFadden 2012). Our present study agrees with some of their conclusions but there are several key differences. Uda et al. (2011) suggest two possible mechanisms by which the *japonicum* and *konojoi* mt gene orders arose. Their favored mechanism involves tandem duplication by slipped-strand mispairing followed by a random loss of genes and inversion by intramitochondrial recombination. This mechanism leads to the *japonicum* gene arrangement first and the *konojoi* arrangement second.

Brockman and McFadden (2012) also lend support to a *japonicum* mt gene arrangement evolving first, but their proposed mechanism of inversions leading to the *japonicum* arrangement cannot explain the creation of the inverted repeat sequences observed in all these taxa. They sequenced the full mitochondrial genome of *Pa. aldersladei* (family Alcyoniidae), discovering the fifth novel gene arrangement in octocorals. Then they proceed to map the five different arrangements onto a phylogeny of the Octocorallia based on two mitochondrial genes (*mutS* and *cox1*) and a nuclear gene (*28S*). Their phylogeny shows that the *japonicum* gene arrangement evolved first, before the *konojoi* arrangement, in the branch leading to the Coralliidae and Paragorgiidae. They present *Paracorallium* (now subsumed into *Corallium*; Ardila et al. 2012), which has the *japonicum* gene arrangement, as the sister branch to *Paragorgia* and *C. konojoi* and *Corallium kishinouyei* (the genus *Pleurocorallium* has been resurrected for these species; Figueroa and Baco 2014), which have the *konojoi* gene arrangement. Furthermore, they show that *Anthomastus* is the sister branch to the Coralliidae and Paragorgiidae clade. And by using gene junction screening, they determine that *A. ritteri* has the presumed ancestral octocoral mitochondrial gene order.

Our analysis agrees with that of Brockman and McFadden (2012) in placing *Anthomastus* as the sister branch to the Paragorgiidae and Coralliidae, but it differs in that the two morphospecies of *Anthomastus* used in our study have the *japonicum* gene arrangement, while the species of *Anthomastus* used by Brockman and McFadden (2012) has the presumed ancestral gene arrangement. Because Brockman and McFadden (2012) only used gene junction screening to determine the mitochondrial gene arrangement of *A. ritteri*, the possibility remains that instead of being an example of conserved mitochondrial gene order, this particular species of *Anthomastus* could have reverted back to the ancestral state as it happened with *S. cauliflora*. So far, every species of octocoral belonging to McFadden et al.’s (McFadden et al. 2006) *Anthomastus*–*Corallium* clade, which also include the Paragorgiidae (Figueroa and Baco 2014), has a derived mitochondrial gene arrangement, except for *A. ritteri* (Brockman and McFadden 2012). Therefore it would be interesting to sequence the full mitochondrial genome of *A. ritteri*, because if it truly has a conserved ancestral mitochondrial gene order then it is likely a basal member of this major octocoral clade.

Further research is needed to determine the evolutionary order of the mitochondrial gene arrangement in this *Anthomastus*–Corallidae–Paragorgiidae clade. Although Uda et al. (2011) and Brockman and McFadden (2012) support a *japonicum* gene arrangement evolving before the *konojoi* arrangement, our present research shows that this is not necessarily the case because each major branch in this clade has its own unique arrangement with possible reversals to ancestral states and with at least one of these arrangements evolving in two independent events. Therefore it is very likely that when the full mitochondrial genomes are sequenced from more members of this clade, more unique gene orders will be found and possibly more reversals to ancestral states will also be identified.

### Mitochondrial Genomes and Higher Level Phylogenies within Cnidaria

The class Anthozoa consists of two subclasses, the Hexacorallia and the Octocorallia (Daly et al. 2007). The monophyly of Anthozoa is well supported by both morphological and molecular phylogenetic reconstructions based on nuclear genes (France et al. 1996; Odorico and Miller 1997; Berntson et al. 1999; Won et al. 2001; Collins 2002; Daly et al. 2007). However, recent studies based on whole mitochondrial genomes disagree with this observation and suggest that Anthozoa is paraphyletic because in their phylogenetic reconstructions, the Octocorallia is more closely related to the Medusozoa than to the Hexacorallia (Shao et al. 2006; Kayal and Lavrov 2008; Lavrov et al. 2008; Park et al. 2012; Kayal et al. 2013). In our phylogenetic reconstruction, where members of the Porifera were included with the Cnidaria, the resulting unrooted phylogeny shows that the Porifera are a sister branch to the Hexacorallia. If this tree is redrawn and rooted by the Porifera, then the resulting phylogeny appears as if the Hexacorallia are the basal branch to the Cnidaria with the Octocorallia branching later, as a sister clade to the Medusozoa. This is the same pattern observed by those studies that suggest that the Anthozoa is paraphyletic (Shao et al. 2006; Kayal and Lavrov 2008; Lavrov et al. 2008; Park et al. 2012; Kayal et al. 2013). This suggests that the close association between the Octocorallia and the Medusozoa is likely an artifact due to the use of Porifera as a root for the Cnidaria. This observation is further supported by our phylogenetic analysis that only included the Octocorallia, Hexacorallia, and Medusozoa. This phylogeny clearly shows that based on whole mitochondrial genomes, no assertion can be made whether the Octocorallia belong to the Hexacorallia or the Medusozoa. Each of these taxa form an independent well-supported branch.

Because of the mismatch in previous studies between phylogenies based on whole mitochondrial genomes compared with nuclear and morphological data (France et al. 1996; Odorico and Miller 1997; Berntson et al. 1999; Won et al. 2001; Collins 2002; Shao et al. 2006; Daly et al. 2007; Kayal and Lavrov 2008; Lavrov et al. 2008; Park et al. 2012; Kayal et al. 2013), we explored the possibility of saturation in the mitochondrial sequences that have been used for Cnidaria. Our phylogenetic reconstruction and substitution saturation analysis show that whole mitochondrial genomes can be used effectively for phylogenetic analyses of the Octocorallia. However, it appears that the utility of mt genomes at higher taxonomic levels is limited (figs. 3 and 4).

It has been shown that when substitution saturation is high, similarity between sequences does not accurately reflect phylogenetic relationships (Steel et al. 1993; Xia et al. 2003; Xia and Lemey 2009). Sequences that have not experienced substantial substitution saturation will show a linear relationship for both transitions and transversions versus sequence divergence; also, transitions will occur more often than transversions (Xia and Xie 2001). This relationship is found in the Octocorallia, but it starts to break down in the Hexacorallia and it deviates even further at higher taxonomic levels with the Anthozoa and Cnidaria. This suggests that at higher taxonomic levels the phylogenetic signal in mitochondrial genomes may be lost due to substitution saturation. The statistical tests proposed by Steel et al. (1993) support this observation. These tests showed that when only the Octocorallia or the Hexacorallia are considered, all the sequences are phylogenetically informative. But, when higher taxonomic levels are considered, such as Anthozoa and Cnidaria, more than 80% of the sequences are no longer phylogenetically informative. This clearly shows that the nucleotide sequences of mitochondrial genomes at the Anthozoan and Cnidarian taxonomic level have experienced full substitution saturation and therefore are no longer phylogenetically informative.

To minimize the problem generated by substitution saturation, nucleotide sequences can be translated into amino acid sequences; then they can be translated back into a nucleotide sequence using a standard genetic code, essentially getting rid of any synonymous substitutions. This was done by Park et al. (2012) when using full mitochondrial genomes to look at Cnidarian evolution using the Porifera as a root. Because the alignment by Park et al. (2012) is not available on an online repository, we used the amino acid alignment from Kayal et al. (2013) and followed the methods of Park et al. (2012) to reverse translate this alignment to a nucleotide alignment. The alignment by Kayal et al. (2013) includes all the sequences used by Park et al. (2012) plus many more obtained in that study. We analyzed this new data set for substitution saturation using the tests developed by Xia et al. (2003) and Steel et al. (1993). Xia’s test showed that the observed saturation index is lower than the critical saturation index if the resulting tree is symmetrical, but it is still higher if the tree is asymmetrical (fig. 4). The phylogeny presented by both Park et al. (2012) and Kayal et al. (2013) is highly asymmetrical, which suggests that despite eliminating synonymous substitutions from the analysis, substitution saturation was still a problem for analyzing the Cnidaria using reverse-translated nucleotide sequences. The inadequacy of these reverse-translated nucleotide sequences for reconstructing the phylogeny of the Cnidaria is further supported by Steel’s test which shows that none of the sequences are phylogenetically informative. Therefore, the nucleotide sequences of mitochondrial genomes should not be used to determine phylogenetic relationships for the Anthozoa or the Cnidaria. Kayal et al. (2013) address the issue of nucleotide saturation by removing the third codon position as well as all codons for arginine, leucine, and serine. Additionally, they use amino acid sequences to reconstruct their phylogeny of Cnidaria using the best evolutionary models available to reduce the effects of saturation. Unfortunately, saturation tests for their nucleotide alignments are not presented and no such tests exist for amino acid alignments. Therefore, although they go through great lengths to compensate for saturation, whether their methods were enough will likely go unanswered until other molecular markers are used to reconstruct the phylogeny of Cnidarians. The dubious association of the Porifera as a sister branch of the Hexacorallia and the resulting appearance of the Octocorallia forming a clade with the Medusozoa could just be an artifact of substitution saturation in the mitochondrial genomes of these taxa. Therefore we recommend that for phylogenetic reconstruction at taxonomic levels higher than subclass within the Cnidaria, nuclear genes will be required, even when whole mitochondrial genomes are available.

## Conclusions

Our phylogenetic reconstruction supports two major clades within the Octocorallia. One includes the Alcyoniina and Holaxonia, while the other divides into two branches, one composed of Pennatulacea and Calcaxonia, and the second with *Anthomastus*, Paragorgiidae, and Coralliidae. Our phylogeny also shows that *Paragorgia* and *Sibogagorgia* are two independent lineages, making the Paragorgiidae a paraphyletic group. We propose that to fix this taxonomic inadequacy, the family Sibogagorgiidae should be resurrected.

Our study is the first to show that in the Octocorallia, mitochondrial gene arrangement is not only diverse but it can evolve back to an ancestral state. This has important implications for genetic studies that use gene boundaries to determine the type of mitochondrial gene arrangement present and then use that information for classification or phylogenetic purposes. Therefore we recommend for future studies of gene rearrangements not to rely exclusively on gene junction screening as it will miss reversals to ancestral states.

Further research is needed to determine the evolutionary order of the mitochondrial gene arrangement in the *Anthomastus*–Corallidae–Paragorgiidae clade. Our study shows that each major branch in this clade has its own unique arrangement with possible reversals to ancestral states and with at least one of these arrangements evolving in two independent events.

Our phylogenetic reconstruction and substitution saturation analysis demonstrates that whole mitochondrial genomes can be used effectively for phylogenetic analyses of the Octocorallia. However, the utility of mt genomes at higher taxonomic levels is limited. Therefore we recommend that for phylogenetic reconstruction at taxonomic levels higher than subclass within the Cnidaria, nuclear genes will be required, even when whole mitochondrial genomes are available.
